# Hydric physiology and ecology of a federally endangered desert lizard

**DOI:** 10.1093/conphys/coae019

**Published:** 2024-05-06

**Authors:** Savannah J Weaver, Ian J Axsom, Lindsay Peria, Tess McIntyre, Justin Chung, Rory S Telemeco, Michael F Westphal, Emily N Taylor

**Affiliations:** Department of Biological Sciences, Bailey College of Science and Mathematics, California Polytechnic State University, San Luis Obispo, Fisher Science, 1 Grand Avenue, San Luis Obispo, CA 93407-0401, USA; Department of Biological Sciences, Bailey College of Science and Mathematics, California Polytechnic State University, San Luis Obispo, Fisher Science, 1 Grand Avenue, San Luis Obispo, CA 93407-0401, USA; Department of Biological Sciences, Bailey College of Science and Mathematics, California Polytechnic State University, San Luis Obispo, Fisher Science, 1 Grand Avenue, San Luis Obispo, CA 93407-0401, USA; Department of Biological Sciences, Bailey College of Science and Mathematics, California Polytechnic State University, San Luis Obispo, Fisher Science, 1 Grand Avenue, San Luis Obispo, CA 93407-0401, USA; Department of Biological Sciences, Bailey College of Science and Mathematics, California Polytechnic State University, San Luis Obispo, Fisher Science, 1 Grand Avenue, San Luis Obispo, CA 93407-0401, USA; Department of Conservation Science, Fresno Chaffee Zoo, 894 W Belmont Avenue, Fresno, CA 93728, USA; Department of Biology, College of Science and Mathematics, California State University, Fresno, 2555 East San Ramon Ave, Fresno, CA 93740, USA; Central Coast Field Office, Bureau of Land Management, 940 2nd Avenue, Marina, CA 93933, USA; Department of Biological Sciences, Bailey College of Science and Mathematics, California Polytechnic State University, San Luis Obispo, Fisher Science, 1 Grand Avenue, San Luis Obispo, CA 93407-0401, USA

**Keywords:** Cutaneous evaporative water loss, *Gambelia sila*, hydration, hydroregulation, osmoregulation, plasma osmolality

## Abstract

Animals can respond to extreme climates by behaviourally avoiding it or by physiologically coping with it. We understand behavioural and physiological thermoregulation, but water balance has largely been neglected. Climate change includes both global warming and changes in precipitation regimes, so improving our understanding of organismal water balance is increasingly urgent. We assessed the hydric physiology of US federally endangered blunt-nosed leopard lizards (*Gambelia sila*) by measuring cutaneous evaporative water loss (CEWL), plasma osmolality and body condition. Measurements were taken throughout their active season, the short period of year when these lizards can be found aboveground. Compared to a more mesic species, *G. sila* had low CEWL which is potentially desert-adaptive, and high plasma osmolality that could be indicative of dehydration. We hypothesized that throughout the *G. sila* active season, as their habitat got hotter and drier, *G. sila* would become more dehydrated and watertight. Instead, CEWL and plasma osmolality showed minimal change for females and non-linear change for males, which we hypothesize is connected to sex-specific reproductive behaviours and changes in food availability. We also measured thermoregulation and microhabitat use, expecting that more dehydrated lizards would have lower body temperature, poorer thermoregulatory accuracy and spend less time aboveground. However, we found no effect of CEWL, plasma osmolality or body condition on these thermal and behavioural metrics. Finally, *G. sila* spends considerable time belowground in burrows, and burrows may serve not only as essential thermal refugia but also hydric refugia.

## Teaser

Endangered blunt-nosed leopard lizards (*Gambelia sila*) live at extremes; they experience severe heat and sparse water resources in their desert environment. We assessed the hydric physiology of these lizards in a drought year and demonstrated relatively low water loss rates and low hydration.

## Introduction

Desert animals live life at the extremes, experiencing severe temperatures and sparse water resources. To avoid such harsh conditions, animals can behaviourally buffer themselves by selecting favourable microhabitats (Sunday *et al.*, 2014) and by limiting their activity to specific times of year (Storey, 2002) or times of day (Abom *et al.*, 2012; DeGregorio *et al.*, 2018). Many animals seek refuge in burrows when under threat of desiccation (Nagy and Medica, 1986; Nagy, 1988; Fishman *et al.*, 1992; Christian *et al.*, 1996; Fuller *et al.*, 2021). However, behavioural responses to climate are only effective when microhabitat heterogeneity is available (Sears *et al.*, 2016). Thus, physiological limits are what ultimately determine the geographic distribution of species (Kearney *et al.*, 2018). There has been a wealth of research on such thermal limits (e.g. Angilletta, 2009; Sinervo *et al.*, 2010; Taylor *et al.*, 2020), but a comparative paucity of research on hydric limits. Climate change is well underway (IPCC, 2021), and hydric costs of thermoregulation are likely the biggest drivers of population declines (Riddell *et al.*, 2019a, 2021). It is imperative that we understand the water balance and dehydration tolerances of organisms to predict how species may or may not cope with climate change. This is especially urgent for threatened and endangered species adapted to arid environments, which are already living under hydric stress.

**Figure 1:**
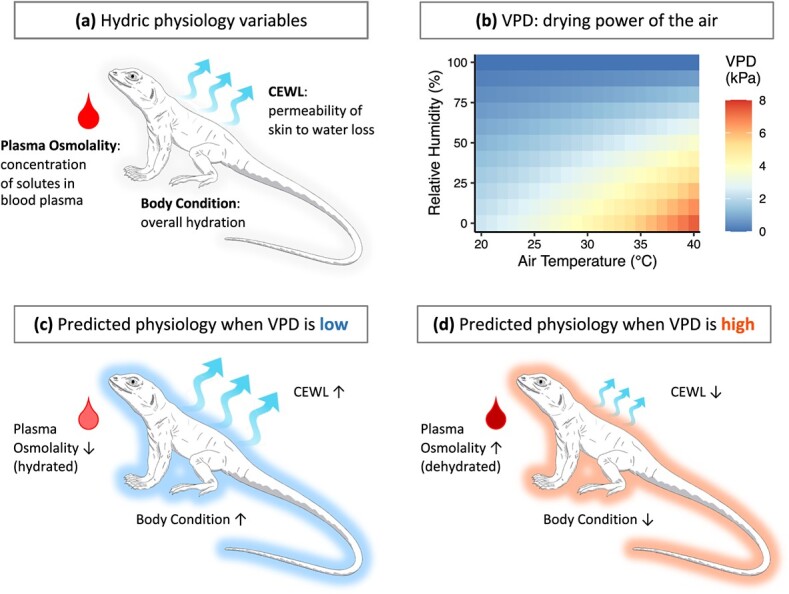
Hydric physiology variables and predictions for this study on *G. sila*. Variables related to hydric physiology (a) include plasma osmolality, CEWL and body condition. VPD (b) quantifies the drying power of the air as a function of both air temperature and humidity. High VPD is desiccating to organisms. We predict that when environmental VPD is low (c), such as in early spring when *G. sila* are emerging from hibernation, they should be able to maintain relatively good hydration, measured as low plasma osmolality and high body condition. Because lizards are hydrated, CEWL should be unrestricted (c). Conversely, we predict that when VPD is high (d), such as during the summer, *G. sila* should become relatively dehydrated, measured as higher plasma osmolality and lower body condition compared to when they are not exposed to desiccating conditions. Because lizards are dehydrated, CEWL should be reduced to aid in water conservation (d). Artwork of *G. sila* by S. Mieko Temple.

Blunt-nosed leopard lizards (*Gambelia sila*) are US federally endangered desert lizards that have been the subject of plenty of thermal physiology and ecology research (e.g. Germano, 2019; Ivey *et al.*, 2020; Gaudenti *et al.*, 2021) but no hydric physiology research. Adult *G. sila* are typically active aboveground from April to July each year, with some opportunistic activity in March, August, September and October (Montanucci, 1965 and 1967; Germano and Williams, 2005). These lizards rely on perennial shrubs, large annual forbs and mammal burrows to behaviourally buffer themselves from mid-day and late summer heat (Ivey *et al.*, 2020; Gaudenti *et al.*, 2021). Despite having ‘apparently no requirement for [free-standing drinking] water’ (Ahlborn, 2000) and lower likelihood of occurrence where rainfall is higher (Stewart *et al.*, 2019), studies suggest that *G. sila* fails to reproduce in drought years (Germano and Williams, 2005; Westphal *et al.*, 2016). So, while these lizards may be able to behaviourally buffer themselves from temperature extremes, water restriction appears detrimental. Accordingly, we set out to characterize the hydric physiology of *G. sila* (Fig. 1).

To assess the typical hydration levels and water loss rates of *G. sila*, we measured cutaneous evaporative water loss (CEWL), plasma osmolality and body condition (Fig. 1A) throughout their active season. Because they should be adapted to hot, dry desert conditions, we hypothesized that *G. sila* would have extremely low water loss rates (Cox and Cox, 2015), and that variation in these rates among individuals would be impacted by individual lizards’ hydration. We predicted that *G. sila* would have higher plasma osmolality (i.e. be less hydrated) and lower CEWL compared to mesic species, which live in comparably water-rich environments. We also expected that the hydric physiology of *G. sila* would change throughout their active season relative to seasonal weather patterns (Davis and DeNardo, 2009, 2010). In the spring when their habitat is relatively lush, *G. sila* should be relatively hydrated (Fig. 1C). But throughout their active season, as their habitat gets hotter and drier, *G. sila* should become dehydrated and maximize water conservation by reducing CEWL (Fig. 1D). In addition to measuring hydric physiology, we used temperature-sensing radio-transmitters to record body temperature and track microhabitat use. As temperatures increase throughout their active season, *G. sila* spends progressively more time belowground (Ivey *et al.*, 2020; Gaudenti *et al.*, 2021), but their decreased surface activity could also be related to dehydration (Davis and DeNardo, 2009). We hypothesized that hydration would constrain thermoregulation, predicting that more dehydrated individuals would maintain lower body temperatures, have poorer thermoregulatory accuracy and spend less time aboveground (Davis and DeNardo, 2009; Sannolo and Carretero, 2019). We also predicted that across individuals, as their habitat got hotter and drier throughout the season, *G. sila* would become dehydrated, leading them to spend more time belowground to conserve water.

## Materials and Methods

### Capture and identification

In the spring and summer of 2021, we used hand-held lassos to capture 79 (32F, 47 M) *G. sila* in the Carrizo Plain National Monument, CA, USA (Table 1). Sample size was constrained only by time and search effort. Each lizard was implanted with a passive integrated transponder (PIT-tag) for identification upon recapture (8 mm MUSICC Integrated Chip; Avid Identification Systems, Inc, Norco, CA, USA), and a subset of lizards (Table 1) were fitted with very high frequency (VHF) temperature-sensing radio transmitter “collars” with 16 cm whip antenna (model BD-2 T; Holohil Systems Ltd, Carp, Ontario, Canada; collar mass 1.9–2.7 g, 3.4–8.6% lizard body mass) as described in Ivey *et al.* (2020). Throughout the study, 13 lizards lost their transmitter collars, and we re-fitted two of those collars to different lizards in May (Table 1). Handling of *G. sila* was approved under Federal Recovery Permit TE-166383, an MOU issued to M.F. Westphal in 2018 by the California Department of Fish and Wildlife, and Cal Poly IACUC #1809.

**Table 1:** Number of *G. sila* for which hydric physiology was evaluated during each measurement period of the study

Measurement period	Newly captured lizards	Recaptured lizards	Total
April 23–25	67 (39)	-	67
May 7–8	11 (2)	26 (21)	37
July 14	1	14 (14)	15
Total	79	40	119

Numbers in parentheses are the subset of lizards that were fitted with radio-transmitter collars.

### Hydric physiology

At each capture or recapture, we collected the following data: lizard mass (Pesola 50–100 g precision scale; ± 0.1 g), snout-vent-length (SVL; ± 1 mm), sex, blood samples either from caudal venipuncture using heparinized needles or from the post-orbital sinus of the right eye using heparinized microhematocrit capillary tubes (Clay Adams, Becton Dickinson, Sparks, MD, USA), and CEWL (± g m^−2^ h^−1^) in 3–5 technical replicates on the same spot on the mid-dorsum using an AquaFlux evaporimeter (model AF200; BioX Systems, London, UK). For each CEWL replicate group, we omitted outliers based on boxplot distributions (*n* = 105 overall) and averaged the remaining values. The AquaFlux measures CEWL as instantaneous movement of water across the skin of a 3-mm diameter area within a closed chamber. When the measurement chamber is pressed against the skin and a closed system is created, accumulated water is removed, then instantaneous water flux is recorded when the reading stabilizes to ±0.02 g m^−2^ h^−1^. The device has high repeatability (Imhof *et al.*, 2014), and it was calibrated following manufacturer guidelines prior to our study and before each set of measurements. Additional explanations and uses of the AquaFlux evaporimeter are available (Imhof *et al.*, 2009; Elkeeb *et al.*, 2010; Lourdais *et al.*, 2017; Weaver *et al.*, 2022). Immediately following CEWL measurements, we recorded lizard body temperature (± 1°C) by inserting a thermocouple into the cloaca (Traceable Pocket-size K-type Thermocouple, model 14–649-81; Thermo Fisher Scientific, Waltham, MA, USA). The evaporimeter also recorded ambient temperature (± 0.1°C) and relative humidity (± 0.1%) at the time of measurement. We used these values to calculate vapour pressure deficit (VPD; Fig. 1B) at the time of CEWL measurement using the equations:

(1) \begin{equation*} \mathrm{VPD}={e}_{\mathrm{s}}-{e}_{\mathrm{a}} \end{equation*}

(2) \begin{equation*} {e}_{\mathrm{s}}=0.611\times \exp \left(\frac{17.502\times T}{T+240.97}\right) \end{equation*}

(3) \begin{equation*} {e}_{\mathrm{a}}={e}_{\mathrm{s}}\times \frac{\mathrm{RH}}{100} \end{equation*}

where *e*_s_ is the saturation vapour pressure (kPa) and *e*_a_ is the actual vapour pressure (kPa), both of ambient air at a given temperature; *T* is temperature (°C) and RH is percent relative humidity (Campbell and Norman, 1998). VPD is a more relevant metric than relative humidity because it assesses the drying power of the air, and therefore the desiccation pressure to which lizards are exposed. A consistent relative humidity value could refer to a wide range of VPDs, depending on temperature (Fig. 1B).

Blood samples were stored on ice for transport to the lab, then centrifuged in a micro-haematocrit centrifuge (model IEC MB; Damon IEC Division, Thermo Fisher Scientific) for 2 minutes. Percent haematocrit (± 1%) was recorded, and plasma was stored in a refrigerator as needed. Haematocrit is the percent red blood cells of blood; the remaining percent is mostly plasma, which is water-based, so the relative quantity of haematocrit could indicate hydration state (see Supplementary Appendix A for results). Plasma osmolality (± 3 mmol kg^−1^) was measured in 1–3 technical replicates on a vapour pressure osmometer (VAPRO, model 5600; Wescor, ELITech, Logan, UT, USA) within 48 hours of blood collection. For triplicate groups with variation exceeding the precision of the osmometer, we removed a replicate if its value did not group with the other two (*n* = 48 overall), and the remaining replicates were averaged. Plasma osmolality refers to the concentration of solutes in the blood; dehydrated animals should have high plasma osmolality.

We compared *G. sila* CEWL and plasma osmolality to those of Western Fence Lizards (*Sceloporus occidentalis*), a mesic lizard measured following the same methods (Weaver *et al.*, 2023). This study presents the first hydric physiology measurements taken on *G. sila.* It is also unclear whether CEWL measurements taken with the AquaFlux evaporimeter are comparable to measurements taken with respirometry–hygrometry or based on mass loss. Thus, our comparison to *S. occidentalis* is the best way to contextualize our novel measurements of *G. sila.* We captured *S. occidentalis* throughout the campus of California Polytechnic State University, San Luis Obispo, CA, USA, ~ 100 km northwest of the study site for *G. sila*. All hydric physiology methods were identical for the two species.

In April and May, we also assessed the gravidity of 19 radio-collared females and the approximate stage of their egg development by palpating the lower abdomen. Gravidity was not assessed for females that were not radio-collared, as they were typically small, and we assumed them to be reproductively inactive. We examined egg development including clutch and egg size with ultrasonography (Sonosite M-Turbo with a HFL50x/15-6Mhz transducer; FUJIFILM Sonosite Inc, Bothell, WA). All measurements and samples were taken within 2–3 hours of capture, with the exception of some females being ultra-sounded the morning following capture. Early in the study, we attempted to supplementally hydrate half of the radio-collared lizards by offering them drinking water prior to release. However, lizards did not drink water, and we observed no effects of this treatment on the hydration of those offered water (see Supplementary Appendix B for results), so we pooled all data for further analysis. Immediately after measurements, lizards were released at their location of capture.

### Thermal ecology and behaviour

We radio-tracked lizards between 07:00 and 18:00 daily from the time they were fitted with collars through mid-July using VHF receivers (TR-8 Handheld Scanning Receivers; Telonics Inc., Mesa, AZ, USA) fitted with antennas (Yagi; Communications Specialists, Orange, CA, USA). Due to dropped transmitter collars and lost signals, we tracked 39 of the 41 collared lizards for a mean ± SD of 45 ± 38 observations over 45 ± 29 days per lizard. We recorded the microhabitat use of each lizard each time. We observed it as one of the following: ‘Burrow’ when lizards were belowground inside burrows engineered by Giant Kangaroo Rats (*Dipodomys ingens*), including lizards that could not be seen and those that were visible deep inside the burrow; ‘Full Shade’ when lizards were under the shade of a shrub and the entire body was shaded; ‘Partial Shade’ when lizards were under shade but part of the body was in the sun; and ‘Open’, when lizards were aboveground in full sun, not under the shade of a shrub, including when at the mouth or apron of a burrow but still visible from above. We also recorded field active body temperature (*T*_b_) with a stationary 3 m tall solar-powered omni-antenna (model RA-6B; Telonics, Mesa, AZ, USA; RemotePro 2.5 W Solar Power System; Tycon Systems, Bluffdale, UT, USA; Eldora 10P solar panel; Vikram Solar Ltd, West Bengal, India) and receiver with data acquisition system (TR-5 Option 320; Telonics) as described in Ivey *et al.* (2020).

At the end of the study in July, we excavated 11 of the estivating lizards and recaptured the 3 lizards that were still active aboveground. Only these 14 lizards survived to the end of the active season and/or still had detectable radio-transmitter signals; there were three other lizards that we tracked to the end of the study, but we were unable to excavate them and remove their collars. Throughout the season, we found 13 dropped transmitter collars, 9 of which may have simply fallen off the lizard and 4 of which showed clear signs of depredation. We lost the signal of 11 transmitter collars, which could be due to dead batteries or lizards that were carried away by avian predators.

### Climate conditions

At our study site, we recorded temperature and relative humidity 1 m inside 11 *D. ingens* burrows every 30 minutes for the duration of the study (HOBO External Temperature/RH Sensor Data Loggers, model MX2302A; Onset Computer, Bourne, MA, USA). We also obtained hourly ambient temperature, relative humidity, wind speed and precipitation from a weather station 3.7 km due east of the study site (station ID CXXC1; Natural Resources Conservation Service, 2021).

### Statistical analysis

#### Hydric physiology

We used linear regression (LR) to assess whether the variables CEWL, plasma osmolality and body condition were interrelated. We also used LRs to quantify the effect of lizard *T*_b_, ambient temperature, and ambient VPD at the time of measurement on CEWL. Lizards ranged from small yearlings (not radio-collared) to large adults (radio-collared; Table 1), so we calculated body condition as scaled mass index (g’; Peig and Green, 2009) using a scaling equation derived from our April mass and SVL measurements. We only made one scaling equation based on the lizards with repeat measurements. SVL did not change throughout the study (linear mixed-effect model (LMM) with individual lizard ID as a random effect: estimate = 0.1, SE = 0.1, t_43_ = 0.7, *P* = 0.5). Body condition is an assessment of relative body mass for a given lizards’ body length; for gravid female lizards, body condition is expected to be high and mostly represent reproductive progress rather than body condition alone. To test differences in plasma osmolality, CEWL and temperature and VPD at the time of CEWL measurement between *G. sila* and *S. occidentalis*, we ran LR for each variable with species as the explanatory variable, then we calculated model-estimated means and confidence intervals.

To assess how hydric physiology changed throughout the *G. sila* active season, we used LMMs to quantify how CEWL, plasma osmolality and body condition differed across measurement periods and between sexes, with individual lizard ID as a random effect to account for repeated measurements (1–3 measurements per lizard). For CEWL, we tested the addition of covariates ambient temperature and VPD at the time of CEWL measurement. To assess whether the likelihood of a female being gravid was related to CEWL, plasma osmolality or body condition, we used generalized linear mixed-effects models (GLMMs) with a binomial distribution and individual lizard ID included as a random effect.

#### Thermal ecology and behaviour

For each lizard, we took a subset of daily *T*_b_ data between the 80 and 90th percentiles, then calculated daily ‘maximum T_b_’ as the mean of that subset. We used the 80–90th percentiles instead of actual daily maximum *T*_b_, and extreme surface body temperature values from each lizard (>2 SD away from individual mean) were omitted (<5% of all points) because the highest temperature values for body surface temperatures tend to be inflated due to the lizard basking and exposing their temperature-sensing radio-transmitter directly to the sun. We calculated thermoregulatory accuracy by subtracting preferred *T*_b_ (data from Ivey *et al.*, 2020; Gaudenti *et al.*, 2021) from each instance of daytime *T*_b_ (between 07:00 and 19:00; as in Ivey *et al.*, 2020) and taking the absolute value (Hertz *et al.*, 1993), with zero representing perfect accuracy. Microhabitat use was calculated for individual lizards and for all radio-tracked lizards overall as the proportion of total observations that were in each microhabitat; we also calculated proportion of time aboveground (in open or shade microhabitats) versus belowground (in burrow microhabitat).

We paired the April and May hydric physiology measurements from each lizard with their average maximum *T*_b_, thermoregulatory accuracy and proportion of time spent aboveground during the 11-day time interval following that hydric physiology measurement period. We ran a LMM on each relationship with individual lizard ID as a random effect. Clustering the data into 11-day time intervals was arbitrary and simply fit with the hydric physiology measurement periods. We followed the same 11-day time interval clustering pattern to look at how microhabitat use and climate changed throughout the active season. To assess whether the probability that a lizard would be found belowground (in Burrow microhabitat) changed throughout the active season, we ran a GLMM with time interval, lizard sex and their interaction as explanatory variables. The GLMM had a binomial distribution and included individual lizard ID as a random effect.

#### Climate conditions

We calculated VPD as described above (Campbell and Norman, 1998) for all recorded temperature and relative humidity values, for both burrow and weather station data. For each climate variable, we used the average daily mean for each 11-day time interval throughout the active season. Daytime and nighttime were calculated separately and defined by sunrise and sunset times at our study site (Global Monitoring Laboratory, 2021). We also used the local weather station to get the annual cumulative winter precipitation from December to March for each year 2018–2021.

#### Software

All statistics and figures were done in R v4.2.2 (R Core Team, 2022) using tidyverse workflow (Wickham, 2022). We used the lm function for linear models; the lmer and glmer functions in the lmerTest package for LMMs and GLMMs (Kuznetsova *et al.*, 2020; Bates *et al*., 2022); the ANOVA function for type 2 sum of squares values with Kenward–Roger degrees of freedom and the emmeans and pairs functions in the emmeans package for model-estimated means, confidence intervals and their pairwise differences (Lenth *et al.*, 2022). Plots were made with ggplot2 (Wickham *et al.*, 2022). Functions without specified packages are from base R. χ^2^ tests are type 2 with a Pearson distribution. Data and code are archived on Zenodo (doi.org/10.5281/zenodo.10530116).

**Figure 2:**
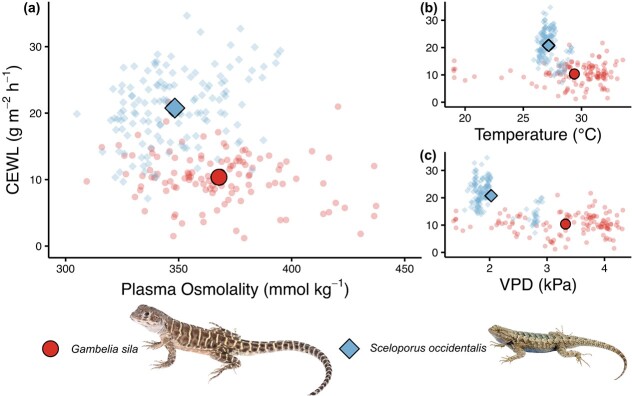
CEWL of desert blunt-nosed leopard lizards (*G. sila*) captured in the Carrizo Plain National Monument, CA, USA versus mesic Western Fence Lizards (*Sceloporus occidentalis*) captured throughout the campus of California Polytechnic State University, San Luis Obispo, CA, USA (Weaver *et al.*, 2023). CEWL is compared relative to hydration (plasma osmolality (a) and ambient temperature (b) and VPD (c) at the time of CEWL measurement. Each small point represents a measurement of an individual lizard taken shortly after their capture. Large points represent model-estimated means. 95% confidence intervals were removed because they were obscured by mean points. CEWL, plasma osmolality and temperature and VPD at the time of measurement were all significantly different between species (all comparisons *p* < 0.0001). Photo of *G. sila* by Robert Hansen. Photo of *S. occidentalis* by Jackson Shedd.

## Results

### Hydric physiology

Compared to more mesic *S. occidentalis*, *G. sila* had an average 50% lower CEWL: 10.4 ± 0.9 g m^−2^ h^−1^ versus 20.8 ± 0.8 g m^−2^ h^−1^ (model-estimated means ±95% confidence interval; t_252_ = −17.4, *P* < 0.0001; Fig. 2; Weaver *et al.*, 2023). *Gambelia sila* were also less hydrated on average, with plasma osmolality 368 ± 2 mmol kg^−1^ compared to 348 ± 2 mmol kg^−1^ for *S. occidentalis* (*t*_252_ = 6.7, *P* < 0.0001; Fig. 2A). Also, we found this difference in CEWL even with *G. sila* being measured at higher temperatures (29.4 ± 0.4°C versus 27.2 ± 0.4°C; *t*_252_ = 7.9, *P* < 0.0001; Fig. 2B) and VPDs (3.3 ± 0.1 kPa versus 2.0 ± 0.1 kPa; *t*_252_ = 17.2, *P* < 0.0001; Fig. 2C).

**Figure 3:**
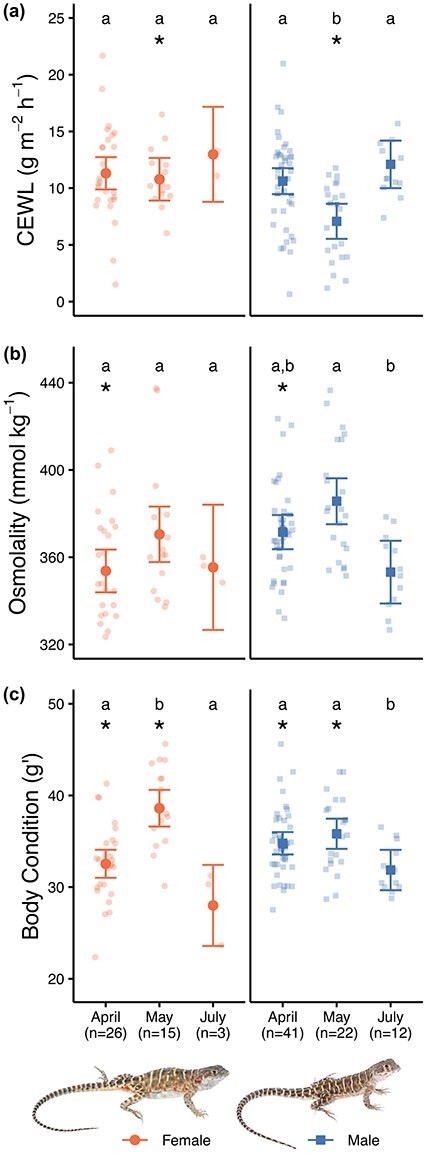
Hydric physiology of *G. sila* differed across measurement periods throughout their active season and based on lizard sex. Hydric physiology was measured as CEWL (a), plasma osmolality (b) and body condition (c; Peig and Green, 2009). Large points with error bars represent model-estimated means ±95% confidence intervals. Each small point represents a measurement on an individual lizard. Sample sizes are noted on the x-axis. Different letters denote significant pairwise differences between measurement periods for a given sex (*P* < 0.0001 for females; *P* < 0.04 for males), and asterisks denote significant pairwise differences between sexes for a given measurement period (*P* < 0.04), based on that linear mixed-effects model (Table 2). These means are on the full dataset from our study, which includes 1–3 measurements on any given lizard; means based only on data from lizards with 2–3 repeat measurements showed the same patterns. Photos of female and male *G. sila* by Robert Hansen.

CEWL, plasma osmolality and body condition of *G. sila* all showed temporal variation, with different patterns based on lizard sex (Table 2). Female lizards experienced no change in CEWL or plasma osmolality throughout the active season (Fig. 3A, B). CEWL for male lizards was lower in May than it was for April or July (Fig. 3A), and plasma osmolality for male lizards was lower in July than in May (Fig. 3B). The relative differences by sex and measurement period in CEWL and plasma osmolality were the same when assessed only in lizards with repeated measurements, with some statistical differences: plasma osmolality also differed between sexes in May (*t*_66_ = −3.8, *P* = 0.0003), and male plasma osmolality differed between April and July (*t*_50_ = 2.5, *P* = 0.04). Covariates temperature and VPD at the time of CEWL measurement had no effect when added to the CEWL model, whether our entire dataset or only repeat measures were used (LMM on full dataset, temperature: SS = 9, *F*_1,110_ = 0.8, *P* = 0.4; VPD: SS = 19, *F*_1,110_ = 1.8, *P* = 0.2; compared to Table 2). On average, the body condition of female lizards was higher in May than in April or July, to be expected due to our inclusion of gravid females, while male lizards had consistent body condition from April to May, then decreased in July (Fig. 3C). When assessed only in lizards with repeated measurements, males again had the same decrease in body condition in July (*t*_51_ = 2.7, *P* = 0.03), female body condition showed the same pattern (Fig. 3C), and the only difference between sexes was body condition in May (*t*_65_ = 2.9, *P* = 0.005). Notably, only three female lizards were recaptured in July, so their statistical results for that month should be interpreted with caution.

**Table 2:** Linear mixed-effect model results for how much of the variation in CEWL, plasma osmolality and body condition of *G. sila* is explained by measurement period (month) throughout the active season, lizard sex and their interaction (Fig. 3)

Hydric response variable	Explanatory variable	SS	*F* statistic _df_	*P* value
	Month	182	8.5_2,77_	< 0.001
CEWL (g m^−2^ h^−1^)	Sex	55	5.2_1,73_	0.03
	Month^*^Sex	47	2.2_2,85_	0.1
	Month	9627	8.3_2,87_	< 0.001
Plasma osmolality (mmol kg^−1^)	Sex	5545	9.6_1,70_	0.003
	Month^*^Sex	770	0.7_2,92_	0.5
	Month	348	16.2_2,71_	< 0.0001
Body condition (g’)	Sex	9	0.8_1,75_	0.4
	Month^*^Sex	138	6.4_2,80_	0.003

SS = partial sum of squares, obtained using type 2 ANOVA with Kenward–Roger degrees of freedom; month = measurement period (April, May or July); Sex = lizard sex (Male, Female); Month^*^Sex = interaction effect.

Of the 19 radio-collared female lizards we ultra-sounded in April and May, 12 were gravid at one or both measurement periods. Each gravid female had 3–5 eggs. The number of eggs a female had was positively correlated with her SVL (*F*_1,6_ = 19.7, *P* = 0.004) and body mass (*F*_1,6_ = 14.8, *P* = 0.008). For the females that were gravid by May, regardless of whether they were gravid in April, CEWL did not change from April to May (*t*_6_ = −0.6, *P* = 0.6). Conversely, plasma osmolality (*t*_6_ = 2.9, *P* = 0.03) and body condition (*t*_6_ = 2.7, *P* = 0.04) both increased from April to May. These changes in gravid females mirror the average change for all female lizards in this study, which includes the 16 small females for which gravidity was not assessed (Fig. 3). The probability of a female being gravid was not affected by their CEWL rates, plasma osmolality or body condition (all *X*^2^ < 1.4, all *P* > 0.2).

**Figure 4:**
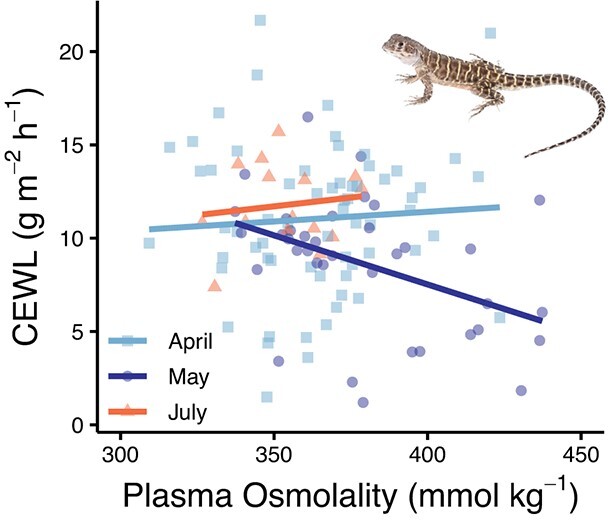
Relationship between CEWL and plasma osmolality of *G. sila* based on measurement period throughout their active season. Each small point is a different lizard at a given time point. Lines represent linear regression (adj-R^2^ = 0.12).

When we assessed the interrelation of hydric physiology metrics, CEWL and plasma osmolality had a relationship, but only for one of the measurement periods. There was a negative relationship in May (estimate = −0.06, SE = 0.03, *t*_110_ = −2.3, *P* = 0.02), but no relationship in April or July (both *t* < 0.6, both *P* > 0.5; Fig. 4). The effect of measurement period (April, May, July) on CEWL was significant (*F*_2,110_ = 4.8, *P* = 0.01; Fig. 4), but the singular effect of plasma osmolality was not (*F*_1,110_ = 1.3, *P* = 0.2), and their interaction effect was marginally non-significant (*F*_2,110_ = 2.8, *P* = 0.07). CEWL could also be explained by a negative relationship with body condition (*F*_1,116_ = 3.9, *P* = 0.05). In turn, variation in plasma osmolality could be explained by a positive relationship with body condition (*F*_1,114_ = 5.3, *P* = 0.02).

### Thermal ecology and behaviour

**Figure 5:**
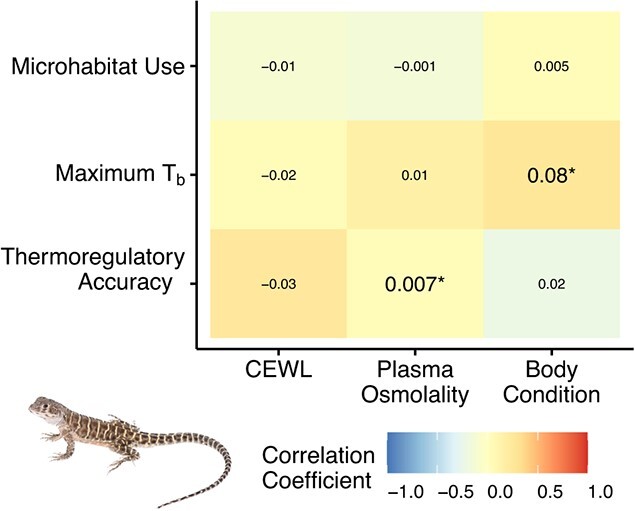
Correlation heatmap of *G. sila* microhabitat use (as the proportion of time spent aboveground), maximum surface body temperatures (*T*_b_) and thermoregulatory accuracy, based on CEWL, plasma osmolality and body condition (Peig and Green, 2009). Colour indicates correlation coefficient. Numbers on the grid represent slope from each linear mixed-effects model. Asterisks denote significant relationships (*P* < 0.05).

The proportion of time lizards spent aboveground was not related to CEWL (*F*_1,3_ = 6.1, *P* = 0.1), plasma osmolality (*F*_1,8_ = 0.5, *P* = 0.5) or body condition (*F*_1,23_ = 0.3, *P* = 0.6). Maximum *T*_b_ was positively related to body condition (*F*_1,41_ = 4.3, *P* = 0.04), but the effect was small (Fig. 5). Maximum *T*_b_ was not related to CEWL (*F*_1,42_ = 0.3, *P* = 0.6) or plasma osmolality (*F*_1,47_ = 3.0, *P* = 0.09). Thermoregulatory accuracy was positively related to plasma osmolality (*F*_1,18_ = 7.3, *P* = 0.01), but the effect was again small (Fig. 5). Thermoregulatory accuracy was not explained by CEWL (*F*_1,48_ = 0.8, *P* = 0.4) or body condition (*F*_1,46_ = 2.6, *P* = 0.1). None of these relationships differed based on lizard sex.

**Figure 6:**
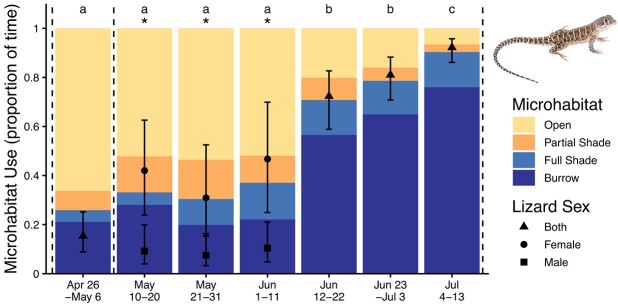
Microhabitat use of *G. sila* throughout their active season, tracked daily between 07:00 and 18:00. Overlaid points with error bars represent model-estimated means ±95% confidence intervals for the probability of being found belowground in burrows, based on the GLMM with a random effect of individual lizard ID. Confidence intervals are asymmetric due to transformation from log-odds to probabilities. Different letters denote differences in probability based only on time interval (*P* < 0.0001). Asterisks denote time intervals when females were more likely to be found belowground than males (*P* < 0.01). Vertical dashed lines show when hydric physiology measurements were taken.

The probability of a lizard being found belowground between 07:00 and 18:00 h differed among time intervals throughout the active season (χ^2^_6_ = 283.7, *P* < 0.0001) and based on sex (χ^2^_1_ = 5.3, *P* = 0.02; interaction: χ^2^_6_ = 20.3, *P* = 0.002). From May 10 to June 11, females were more likely to be in burrows than males (Fig. 6). Microhabitat use did not differ between sexes for any other time interval (*P* > 0.06). For both sexes, the probability of being belowground during the day increased throughout the active season (Fig. 6). When lizards were aboveground early in the season, they used partial shade more than full shade, whereas later in the season, they used full shade more than partial shade (Fig. 6).

### Climate conditions

**Figure 7:**
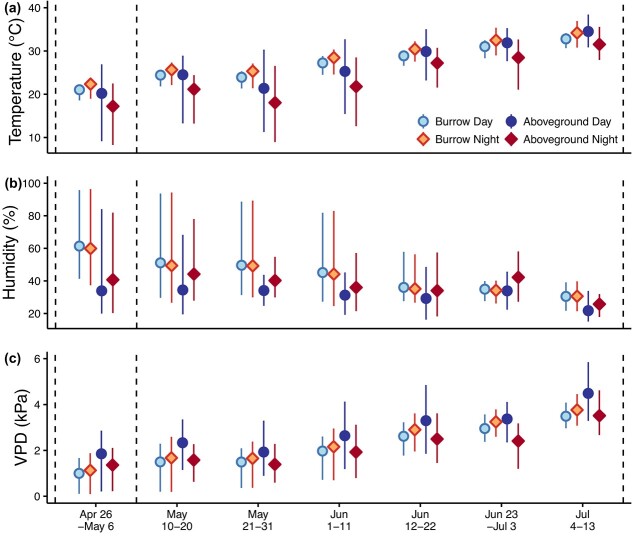
Burrow microhabitat versus local aboveground climate where *G. sila* were studied. Temperature (a) and relative humidity (b) were recorded by a local weather station near the study site and by data loggers placed 1 m into burrows at the study site. VapoVPD (c) was calculated based on temperature and relative humidity values (Eq 1–3; Campbell and Norman, 1998). Points represent average daily mean values ± minimum and maximum daily mean for each time interval. Vertical dashed lines show when hydric physiology measurements were taken. Burrow temperatures were warmer at night than during the day, as expected based on well-described time lags in the thermal diel cycle deep below ground (Telemeco *et al.*, 2016).

Throughout the active season, temperature and VPD increased, and relative humidity decreased for burrow microclimates and for local ambient climate (Fig. 7). Wind speed did not consistently and directionally change throughout the season. Cumulative local winter precipitation was 99 mm in 2018, 193 mm in 2019, 114 mm in 2020 and 74 mm in our study year, 2021. The local winter rainfall immediately prior to the *G. sila* active season of our study in 2021 was 55% of the prior 3-year average.

## Discussion

### Hydric physiology

We found that *G. sila* are watertight and maintain higher plasma osmolality relative to more mesic lizards. On average, CEWL for *G. sila* was much lower than for more-mesic *S. occidentalis* measured using the same methods (Fig. 2; Weaver *et al.*, 2023). We cannot directly compare them to additional studies due to differences in measurement methods and ambient conditions, and we emphasize the need of a comparison of the different methods for measuring evaporative water loss. High temperature and VPD lead to increased CEWL at acute time scales of minutes to hours (Warburg, 1965; Cooper and Withers, 2008; Riddell *et al.*, 2019b; Vicenzi *et al.*, 2021; Davis *et al.*, 2024), but *G. sila* had lower CEWL than *S. occidentalis* even with being measured at higher temperatures and VPDs (Fig. 2B, C), suggesting that *G. sila* have adaptively low CEWL that helps them reduce water loss to their arid environment. This aligns with the hypothesis that decreased skin permeability to water loss is necessary for inhabiting arid environments (Cox and Cox, 2015).

We also observed plasma osmolality values of 320–440 mmol kg^−1^ for *G. sila* (Figs 2A, 3B). Another desert lizard, the Gila Monster (*Heloderma suspectum*), had plasma osmolality ~ 290 mmol kg^−1^ when hydrated and ~ 360 mmol kg^−1^ when dehydrated (Davis and DeNardo, 2007), suggesting that most of the *G. sila* we measured were dehydrated to some degree. Dehydration was to be expected given that our study occurred during a drought year, with winter precipitation approximately half the prior 3-year average (US Drought Monitor, 2021). Not only do *G. sila* tolerate relatively high plasma osmolality compared to another desert lizard, but the inter-individual variation we measured in plasma osmolality suggests that they may also tolerate extreme fluctuations in plasma osmolality. Tolerance of variability in plasma osmolality likely benefits *G. sila* because resources in their habitat are variable and unpredictable. However, this tolerance may also necessitate potentially costly coping mechanisms. The adaptations and mechanisms that animals use to maintain plasma osmolality within a narrow range are widely recognized (Willmer *et al.*, 2005), but more effort should go into understanding how animals, especially desert reptiles, tolerate large changes in and high values of, plasma osmolality.

We hypothesized that throughout the active season for *G. sila*, as spring shifted to summer and their habitat got hotter and drier (Fig. 7), these lizards would become progressively more dehydrated and more watertight. Instead, we observed relatively consistent CEWL, plasma osmolality and body condition (Fig. 3). The observed patterns may be related to prey abundance and the differences in life history between males and females. After emergence in April, lizards can gorge themselves on relatively abundant arthropod prey, improving body condition and hydration. Although the abundance of arthropods was lower during this study than other years due to the drought, arthropods are generally most abundant in April and early May (T. McIntyre, S.J. Weaver and E.N. Taylor, unpublished data). As the season progresses, arthropods begin to disappear, and by July, lizards have lost some body condition (Fig. 3C), potentially due to the lack of food. Despite the assumed hydric gain of feeding on arthropods, male *G. sila* had lower CEWL and higher plasma osmolality in May than in April and July (Fig. 3A, B), and no change in body condition from April to May (Fig. 3C). However, the increased osmolality in May could be due to the hydric cost of feeding and digestion (Chabaud *et al.*, 2023). Given that males must search for mates and defend territories (Montanucci, 1965 and 1967; Tollestrup, 1983), and that this was a drought year (US Drought Monitor, 2021) with dry vegetation and relatively low arthropod abundance, male *G. sila* might have used more water than they could replenish with their diet, resulting in the increased plasma osmolality and decreased CEWL in May (Fig. 3A, B). The ensuing drop in plasma osmolality for males later in the summer (Fig. 3B) was surprising. Considering that male *G. sila* also decreased body condition from May to July (Fig. 3C), they could be catabolising muscle to maintain hydration (Brusch *et al.*, 2018). The changes could also be due to a lack of ingesting solutes from the decrease in arthropod prey late in the season, but mesic lizards are unable to maintain or improve hydration without drinking water (Chabaud *et al.*, 2023). We cannot attribute our findings to either of these mechanisms, but *G. sila* seems to have adaptations that help maintain and even improve hydration.

Changes in body condition for females could be due to the additive effects of feeding and egg development: as more resources are put towards egg development between April and May, females become heavy with eggs. Then, by July, females lay eggs and lose mass (Fig. 3C). Feeding should result in water gain, and egg development should result in water loss, with the two counteracting each other when it comes to hydration, potentially leading to the consistent plasma osmolality and CEWL values observed for females (Fig. 3A, B). Alternatively, it is possible that females were not eating, and not allocating water to their eggs. Water restriction did not affect reproductive output in viviparous lizards (Dupoué *et al.*, 2017), suggesting that female *G. sila* likely develops and lay eggs despite drought, such as during our study. However, dehydrated females have dehydrated eggs (Brusch *et al.*, 2019) and less viable offspring (Dupoué *et al.*, 2017), so hatching and/or hatchling success, rather than maternal reproductive investment, may be the mechanism underlying lack of recruitment for *G. sila* in drought years (Westphal *et al.*, 2016).

Unsurprisingly, each of the hydric physiology metrics we assessed was correlated. Plasma osmolality and CEWL were related, but the direction of that relationship differed depending on when hydric physiology was assessed. In April and July, there was a slight positive relationship, while in May there was a strong negative relationship (Fig. 4). The difference in this correlation among time periods could relate to the changes in CEWL and plasma osmolality throughout the active season, especially for males, which made up most of our sample size (Fig. 3). This negative relationship suggests that when lizards are dehydrated, measured as high plasma osmolality, they become more watertight, reducing CEWL (Figs 3A, 3B, 4), similar to observations in toads (Anderson *et al.*, 2017; Senzano and Andrade, 2018). Dehydration minimizes the ability to evaporatively cool in another desert lizard (DeNardo *et al.*, 2004), but evaporative water loss could also be reduced to maintain hydration (Gerson *et al.*, 2019). We cannot say based on our study whether *G. sila* reduces CEWL to maintain hydration or have decreased CEWL due to dehydration. However, the sex differences we found might suggest that because males are more active (Fig. 6), they become dehydrated (Fig. 3B) and reduced CEWL follows (Figs. 3A, 4). This emphasizes the need to investigate how and why different metrics of organismal water balance are interrelated.

### Thermal ecology

Although there was variability in hydric physiology across individual lizards and measurement periods (Figs 3, 4), hydric physiology did not meaningfully correlate with any thermal ecology metrics (Fig. 5). We predicted that more hydrated lizards (with low plasma osmolality) would maintain higher body temperatures, have better thermoregulatory accuracy and spend more time aboveground (Ladyman and Bradshaw, 2003; Davis and DeNardo, 2009; Sannolo and Carretero, 2019), but we were unable to detect any hydric constraints on thermoregulation and microhabitat use. Basking and maintaining activity may be much more important to these heliothermic lizards than hydric homeostasis. Their tolerance of dehydration, shown in their high plasma osmolality values, could be due to the necessity of foraging and mating within their short active season. Rather than hydration constraining thermoregulation, as seen in other lizards (Davis and DeNardo, 2009; Sannolo and Carretero, 2019; Rozen-Rechels *et al.*, 2020b, 2020a), *G. sila* might tolerates dehydration to maintain thermoregulation. However, if *G. sila* are adapted to readily tolerate dehydration, it is even more puzzling why we did not observe somewhat linear decreases in CEWL (Fig. 3A) and increases in plasma osmolality (Fig. 3B) throughout the active season while *G. sila* were spending time aboveground during the day (Fig. 6) and their habitat got hotter and drier (Fig. 7).

An alternative explanation for why we detected no relationships between hydric physiology and thermal ecology (Fig. 5) is that this is an artefact of all lizards in this study being dehydrated. All radio-tracked lizards may have been spending equally high proportions of time in burrows due to dehydration (Fig. 6). Our entire study could represent one end of the relationship we hypothesized that dehydration constrains thermoregulation (Rozen-Rechels *et al.*, 2019). In our study, most of the lizards were likely dehydrated compared to wetter years, and thus potentially thermally constrained. But perhaps if hydrated lizards were compared to dehydrated lizards, we would observe the predicted hydric constraints on thermal ecology.

### Behaviour and climate

At the beginning of their active season, *G. sila* spent most of their time aboveground. Then in mid-June, they more than doubled the amount of daytime spent in burrows, and they continued to increase the proportion of time spent belowground through the end of the study (Fig. 6). The increased proportion of time spent in burrows (Fig. 6) qualitatively parallels the increased heat and dryness as the active season goes on (Fig. 7). This shift could be due to universally decreased activity, or a shift to more crepuscular activity, which we did not survey for. Other studies posit that this microhabitat use pattern, observed at both seasonal and daily scales, is for thermoregulation (Ivey *et al.*, 2020; Gaudenti *et al.*, 2021). Indeed, the sudden increase in time spent belowground in burrows corresponded to an increase in local aboveground temperatures (Figs. 6, 7A). However, VPD, a measure of the desiccation pressure of the air based on both temperature and water, increased similarly (Fig. 7). Compared to local aboveground daytime conditions, VPD was consistently lower at night aboveground and at all times in burrows (Fig. 7C). While lizards certainly use burrows to avoid thermal extremes aboveground, our data indicate that burrows are likely helpful to *G. sila* water balance. By selecting less-desiccating microhabitats, *G. sila* can decrease the amount of water lost to their environment (Seebacher and Alford, 2002; Dezetter *et al.*, 2022). From May 10 to June 11, females spent more time in burrows than males (Fig. 6), likely related to egg laying behaviour, and possibly a factor that led to less change in CEWL and plasma osmolality throughout the active season for females compared to males (Figs. 3A, 3B). Increased time spent in burrows (Fig. 6) could be proactive to preserve hydration, or it could be reactive to dehydration. Microhabitat selection could be equally or more important for water balance as it is for thermoregulation (Guillon *et al.*, 2014; Pintor *et al.*, 2016; Lourdais *et al.*, 2017), and understanding the hydric needs of these endangered lizards will be essential for their conservation.

## Conclusion

Our assessment of *G. sila* water balance could either show that these lizards are exceptionally desert-adapted, or that they are dangerously water-stressed. These desert lizards have relatively low CEWL (Fig. 2), and more dehydrated lizards tend to be more watertight (Fig. 4), suggesting that *G. sila* are adapted to conserve water. CEWL and plasma osmolality were relatively consistent throughout these lizards’ active season (Fig. 3A, B), despite their environment getting hotter and drier (Fig. 7), and we detected no effect of hydric physiology on thermoregulation or microhabitat selection (Fig. 5). Our data indicate that either *G. sila* tolerate dehydration to maintain their usual behaviour or all *G. sila* in this study were equally dehydrated, and thus equally thermally and behaviourally constrained. In either case, there is certainly a limit to this dehydration tolerance; a single drought year may not be detrimental, but repeated drought years are more likely to surpass their limits (Dodd, 1993; Selwood *et al.*, 2015). Given that we conducted our study in a dry year amidst a drought (US Drought Monitor, 2021), we do not know what CEWL and plasma osmolality would be for hydrated *G. sila*, so future studies should assess these values during a wetter year. Alternatively, experiments could be carried out with captive *G. sila* or a surrogate crotaphytid species to explicitly measure hydration, test dehydration tolerance and assess the effects of dehydration on behaviour (e.g. Dezetter *et al.*, 2022). Although *G. sila* did not drink when we offered them water, supplemental feeding of gut-loaded arthropods could be more likely to lead to changes in hydration and behaviour (but see Chabaud *et al.*, 2023). Or, misting enclosures, rather than providing standing water, could more closely simulate their natural water resources and be more likely to lead to drinking.

Hydration is clearly an important variable impacting the ecology, physiology and behaviour of arid-adapted animals like *G. sila*. Supplemental hydration could be an effective conservation intervention to mitigate the potential negative impacts of dehydration, especially during severe drought years. However, the feasibility of such an intervention is questionable. For small populations on the brink of extinction such as *G. sila*, individuals could be captured and provided with food and water inside enclosures for a short period of time, then released. But, the benefits of hydration would need to be weighed against the potential negative effects of capturing stress. Continuing to collect data on the hydric and thermal physiology and ecology of *G. sila* will aid their conservation by improving our understanding of their ecophysiological limits and enabling effective mechanistic distribution models. That knowledge will in turn inform where conservation breeding programs could (re)establish populations and where land restoration projects could be beneficial. We must understand water balance to understand the effects of climate change on organisms and to determine worthwhile, effective actions to mitigate those effects.

## Supplementary Material

Web_Material_coae019

## Data Availability

All raw data and code for this project are publicly archived on Zenodo (doi.org/10.5281/zenodo.10530116).

## References

[ref1] Abom R , Bell K, Hodgson L, Schwarzkopf L (2012) Moving day and night: highly labile diel activity patterns in a tropical snake. Biotropica 44: 554–559. 10.1111/j.1744-7429.2012.00853.x.

[ref2] Ahlborn G (ed) (2000) Blunt-nosed Leopard Lizard (*Gambelia sila*). In California Wildlife Habitat Relationships System. California Interagency Wildlife Task Group, California Department of Fish and Wildlife, Sacramento, CA

[ref3] Anderson RCO , Bovo RP, Eismann CE, Menegario AA, Andrade DV (2017) Not good, but not all bad: dehydration effects on body fluids, organ masses, and water flux through the skin of *Rhinella schneideri* (Amphibia, Bufonidae). Physiol Biochem Zool 90: 313–320. 10.1086/690189. 28384420

[ref4] Angilletta MJ (2009) Thermal Adaptation: A Theoretical and Empirical Synthesis. Oxford University Press, Oxford

[ref5] Bates D , Maechler M, Bolker B, Walker S, Christensen RHB, Singmann H, Dai B, Scheipl F, Grothendieck G, Green P et al. (2022) lme4: linear mixed-effects models using “Eigen” and S4.

[ref6] Brusch GA , Heulin B, DeNardo DF (2019) Dehydration during egg production alters egg composition and yolk immune function. Comp Biochem Physiol A Mol Integr Physiol 227: 68–74. 10.1016/j.cbpa.2018.10.006. 30300746

[ref7] Brusch GA , Lourdais O, Kaminsky B, DeNardo DF (2018) Muscles provide an internal water reserve for reproduction. Proc Biol Sci 285: 20180752. 10.1098/rspb.2018.0752. 30051850 PMC6030541

[ref8] Campbell GS , Norman JM (1998) An Introduction to Environmental Biophysics, Ed2nd. Springer, New York, NY

[ref9] Chabaud C , Brusch GA, Pellerin A, Lourdais O, Le Galliard J-F (2023) Prey consumption does not restore hydration state but mitigates the energetic costs of water deprivation in an insectivorous lizard. J Exp Biol 226: jeb246129. 10.1242/jeb.246129. 37577990

[ref10] Christian K , Green B, Kennett R (1996) Some physiological consequences of estivation by freshwater crocodiles, *Crocodylus johnstoni*. J Herpetol 30: 1–9. 10.2307/1564699.

[ref11] Cooper CE , Withers PC (2008) Allometry of evaporative water loss in marsupials: implications of the effect of ambient relative humidity on the physiology of brushtail possums (*Trichosurus vulpecula*). J Exp Biol 211: 2759–2766. 10.1242/jeb.019463. 18723532

[ref12] Cox CL , Cox RM (2015) Evolutionary shifts in habitat aridity predict evaporative water loss across squamate reptiles. Evolution 69: 2507–2516. 10.1111/evo.12742. 26227547

[ref13] Davis CG , Weaver SJ, Taylor EN (2024) *Cutaneous evaporative water loss in lizards changes immediately with temperature*. 10.5281/zenodo.10574237. 38728691

[ref14] Davis JR , DeNardo DF (2007) The urinary bladder as a physiological reservoir that moderates dehydration in a large desert lizard, the Gila Monster *Heloderma suspectum*. J Exp Biol 210: 1472–1480. 10.1242/jeb.003061. 17401130

[ref15] Davis JR , DeNardo DF (2009) Water supplementation affects the behavioral and physiological ecology of Gila monsters (*Heloderma suspectum*) in the Sonoran Desert. Physiol Biochem Zool 82: 739–748. 10.1086/605933. 19799522

[ref16] Davis JR , DeNardo DF (2010) Seasonal patterns of body condition, hydration state, and activity of Gila monsters (*Heloderma suspectum*) at a Sonoran Desert site. J Herpetol 44: 83–93. 10.1670/08-263.1.

[ref17] DeGregorio BA , Ravesi M, Sperry JH, Tetzlaff SJ, Josimovich J, Matthews M, Kingsbury BA (2018) Daily and seasonal activity patterns of the massasauga (*Sistrurus catenatus*): an automated radio-telemetry study. Herpetol Conserv Biol 13: 10–16.

[ref18] DeNardo DF , Zubal TE, Hoffman TCM (2004) Cloacal evaporative cooling: a previously undescribed means of increasing evaporative water loss at higher temperatures in a desert ectotherm, the Gila Monster *Heloderma suspectum*. J Exp Biol 207: 945–953. 10.1242/jeb.00861. 14766953

[ref19] Dezetter M , Le Galliard J-F, Lourdais O (2022) Behavioural hydroregulation protects against acute effects of drought in a dry-skinned ectotherm. Oecologia 201: 355–367. 10.1007/s00442-022-05299-1. 36564481

[ref20] Dodd CK (1993) Cost of living in an unpredictable environment: the ecology of striped newts *Notophthalmus perstriatus* during a prolonged drought. Copeia 1993: 605–614. 10.2307/1447221.

[ref21] Dupoué A , Galliard J-FL, Josserand R, DeNardo DF, Decencière B, Agostini S, Haussy C, Meylan S (2017) Water restriction causes an intergenerational trade-off and delayed mother–offspring conflict in a viviparous lizard. Funct Ecol 32: 676–686. 10.1111/1365-2435.13009.

[ref22] Elkeeb R , Hui X, Chan H, Tian L, Maibach HI (2010) Correlation of transepidermal water loss with skin barrier properties in vitro: comparison of three evaporimeters. Skin Res Technol 16: 9–15. 10.1111/j.1600-0846.2009.00406.x. 20384878

[ref23] Fishman AP , Galante RJ, Winokur A, Pack AI (1992) Estivation in the African lungfish. Proc Am Philos Soc 136: 61–72.

[ref24] Fuller A , Mitchell D, Maloney SK, Hetem RS, Fonsêca VFC, Meyer LCR, van de Ven TMFN, Snelling EP (2021) How dryland mammals will respond to climate change: the effects of body size, heat load and a lack of food and water. J Exp Biol 224: jeb238113. 10.1242/jeb.238113. 33627465

[ref25] Gaudenti N , Nix E, Maier P, Westphal MF, Taylor EN (2021) Habitat heterogeneity affects the thermal ecology of an endangered lizard. Ecol Evol 11: 14843–14856. 10.1002/ece3.8170. 34765145 PMC8571645

[ref26] Germano DJ (2019) Activity and thermal biology of blunt-nosed leopard lizards (*Gambelia sila*) in the San Joaquin Desert of California. West N Am Nat 79: 428. 10.3398/064.079.0311.

[ref27] Germano DJ , Williams DF (2005) Population ecology of blunt-nosed leopard lizards in high elevation foothill habitat. J Herpetol 39: 1–18.

[ref28] Gerson AR , McKechnie AE, Smit B, Whitfield MC, Smith EK, Talbot WA, McWhorter TJ, Wolf BO (2019) The functional significance of facultative hyperthermia varies with body size and phylogeny in birds. Funct Ecol 33: 597–607. 10.1111/1365-2435.13274.

[ref29] Global Monitoring Laboratory: Earth System Research Laboratories (2021) Global Radiation and Aerosols: Solar Calculator. National Oceanic and Atmospheric Administration. https://gml.noaa.gov/grad/solcalc/ (2023 January 6, date accessed).

[ref30] Guillon M , Guiller G, DeNardo DF, Lourdais O (2014) Microclimate preferences correlate with contrasted evaporative water loss in parapatric vipers at their contact zone. Can J Zool 92: 81–86. 10.1139/cjz-2013-0189.

[ref31] Hertz PE , Huey RB, Stevenson RD (1993) Evaluating temperature regulation by field-active ectotherms: the fallacy of the inappropriate question. Am Nat 142: 796–818. 10.1086/285573. 19425957

[ref32] Imhof B , Xiao P, Angelova-Fischer I (2014) TEWL, closed-chamber methods: AquaFlux and VapoMeter. In E Berardesca, HI Maibach, K-P Wilhelm, eds, Non Invasive Diagnostic Techniques in Clinical Dermatology. Springer, Berlin, Heidelberg, pp. 345–352.

[ref33] Imhof RE , Jesus MEPD, Xiao P, Ciortea LI, Berg EP (2009) Closed-chamber transepidermal water loss measurement: microclimate, calibration and performance. Int J Cosmet Sci 31: 97–118. 10.1111/j.1468-2494.2008.00476.x. 19175433

[ref34] Intergovernmental Panel on Climate Change (IPCC) (2021) Climate Change 2021: The Physical Science Basis (No. 6).

[ref35] Ivey KN , Cornwall M, Crowell H, Ghazian N, Nix E, Owen M, Zuliani M, Lortie CJ, Westphal M, Taylor E (2020) Thermal ecology of the federally endangered blunt-nosed leopard lizard (*Gambelia sila*). Conserv Physiol 8: 1–11. 10.1093/conphys/coaa014. PMC7047230 33649711

[ref37] Kearney MR , Munns SL, Moore D, Malishev M, Bull CM (2018) Field tests of a general ectotherm niche model show how water can limit lizard activity and distribution. Ecological monographs 88: 672–693. 10.1002/ecm.1326.

[ref38] Kuznetsova A , Brockhoff PB, Christensen RHB, Jensen SP (2020) lmerTest: tests in linear mixed effects models.

[ref39] Ladyman M , Bradshaw D (2003) The influence of dehydration on the thermal preferences of the Western Tiger Snake, *Notechis scutatus*. J Comp Physiol B 173: 239–246. 10.1007/s00360-003-0328-x. 12743727

[ref40] Lenth RV , Buerkner P, Giné-Vázquez I, Herve M, Jung M, Love J, Miguez F, Riebl H, Singmann H (2022) Emmeans: estimated marginal means, aka least-squares means.

[ref41] Lourdais O , Dupoué A, Guillon M, Guiller G, Michaud B, DeNardo DF (2017) Hydric “costs” of reproduction: pregnancy increases evaporative water loss in the snake *Vipera aspis*. Physiol Biochem Zool 90: 663–672. 10.1086/694848. 29068263

[ref42] Montanucci RR (1965) Observations on the San Joaquin leopard lizard, *Crotaphytus wislizenii silus* Stejneger. Herpetologica 21: 270–283.

[ref43] Montanucci RR (1967) Further studies on leopard lizards, *Crotaphytus wislizenii*. Herpetologica 23: 119–126.

[ref44] Nagy KA (1988) Seasonal patterns of water and energy balance in desert vertebrates. J Arid Environ 14: 201–210. 10.1016/S0140-1963(18)31088-7.

[ref45] Nagy KA , Medica PA (1986) Physiological ecology of desert tortoises in southern Nevada. Herpetologica 42: 73–92.

[ref46] Natural Resources Conservation Service (2021) Cochora Ranch Weather Conditions. University of Utah Meso West. https://mesowest.utah.edu/cgi-bin/droman/meso_base_dyn.cgi?stn=cxxc1 (2023 April 1, date accesed).

[ref47] Peig J , Green AJ (2009) New perspectives for estimating body condition from mass/length data: the scaled mass index as an alternative method. Oikos 118: 1883–1891. 10.1111/j.1600-0706.2009.17643.x.

[ref48] Pintor AFV , Schwarzkopf L, Krockenberger AK (2016) Hydroregulation in a tropical dry-skinned ectotherm. Oecologia 182: 925–931. 10.1007/s00442-016-3687-1. 27384338

[ref50] R Core Team (2022) R: a language and environment for statistical computing.

[ref51] Riddell EA , Iknayan KJ, Hargrove L, Tremor S, Patton JL, Ramirez R, Wolf BO, Beissinger SR (2021) Exposure to climate change drives stability or collapse of desert mammal and bird communities. Science 371: 633–636. 10.1126/science.abd4605. 33542137

[ref52] Riddell EA , Iknayan KJ, Wolf BO, Sinervo B, Beissinger SR (2019a) Cooling requirements fueled the collapse of a desert bird community from climate change. Proc Natl Acad Sci 116: 21609–21615. 10.1073/pnas.1908791116. 31570585 PMC6815107

[ref53] Riddell EA , Roback EY, Wells CE, Zamudio KR, Sears MW (2019b) Thermal cues drive plasticity of desiccation resistance in montane salamanders with implications for climate change. Nat Commun 10: 4091. 10.1038/s41467-019-11990-4. 31501425 PMC6733842

[ref54] Rozen-Rechels D , Badiane A, Agostini S, Meylan S, Le Galliard J-F (2020a) Water restriction induces behavioral fight but impairs thermoregulation in a dry-skinned ectotherm. Oikos 129: 572–584. 10.1111/oik.06910.

[ref55] Rozen-Rechels D , Dupoué A, Lourdais O, Chamaillé-Jammes S, Meylan S, Clobert J, Galliard J-FL (2019) When water interacts with temperature: ecological and evolutionary implications of thermo-hydroregulation in terrestrial ectotherms. Ecol Evol 9: 10029–10043. 10.1002/ece3.5440. 31534711 PMC6745666

[ref56] Rozen-Rechels D , Dupoué A, Meylan S, Qitout K, Decencière B, Agostini S, Le Galliard J-F (2020b) Acclimation to water restriction implies different paces for behavioral and physiological responses in a lizard species. Physiol Biochem Zool 93: 160–174. 10.1086/707409. 32031477

[ref57] Sannolo M , Carretero MA (2019) Dehydration constrains thermoregulation and space use in lizards. PloS One 14: e0220384. 10.1371/journal.pone.0220384. 31344149 PMC6657907

[ref58] Sears MW , Angilletta MJ, Schuler MS, Borchert J, Dilliplane KF, Stegman M, Rusch TW, Mitchell WA (2016) Configuration of the thermal landscape determines thermoregulatory performance of ectotherms. Proc Natl Acad Sci 113: 10595–10600. 10.1073/pnas.1604824113. 27601639 PMC5035910

[ref59] Seebacher F , Alford RA (2002) Shelter microhabitats determine body temperature and dehydration rates of a terrestrial amphibian (*Bufo marinus*). J Herpetol 36: 69–75. 10.1670/0022-1511(2002)036[0069:SMDBTA]2.0.CO;2.

[ref60] Selwood KE , Clarke RH, Cunningham SC, Lada H, McGeoch MA, Mac Nally R (2015) A bust but no boom: responses of floodplain bird assemblages during and after prolonged drought. J Anim Ecol 84: 1700–1710. 10.1111/1365-2656.12424. 26179338

[ref61] Senzano LM , Andrade DV (2018) Temperature and dehydration effects on metabolism, water uptake and the partitioning between respiratory and cutaneous evaporative water loss in a terrestrial toad. J Exp Biol 221: jeb188482. 30385484 10.1242/jeb.188482

[ref62] Sinervo B , Méndez-de-la-Cruz F, Miles DB, Heulin B, Bastiaans E, Cruz MV-S, Lara-Resendiz R, Martínez-Méndez N, Calderón-Espinosa ML, Meza-Lázaro RN et al. (2010) Erosion of lizard diversity by climate change and altered thermal niches. Science 328: 894–899. 10.1126/science.1184695. 20466932

[ref63] Stewart JAE , Butterfield HS, Richmond JQ, Germano DJ, Westphal MF, Tennant EN, Sinervo B (2019) Habitat restoration opportunities, climatic niche contraction, and conservation biogeography in California’s San Joaquin Desert. PloS One 14: e0210766. 10.1371/journal.pone.0210766. 30645624 PMC6333358

[ref64] Storey KB (2002) Life in the slow lane: molecular mechanisms of estivation. Comp Biochem Physiol A Mol Integr Physiol 133: 733–754. 10.1016/S1095-6433(02)00206-4. 12443930

[ref65] Sunday JM , Bates AE, Kearney MR, Colwell RK, Dulvy NK, Longino JT, Huey RB (2014) Thermal-safety margins and the necessity of thermoregulatory behavior across latitude and elevation. Proc Natl Acad Sci 111: 5610–5615. 10.1073/pnas.1316145111. 24616528 PMC3992687

[ref66] Taylor EN , Diele-Viegas LM, Gangloff EJ, Hall JM, Halpern B, Massey MD, Rödder D, Rollinson N, Spears S, Sun B et al. (2020) The thermal ecology and physiology of reptiles and amphibians: a user’s guide. J Exp Zool A 335: 13–44. 10.1002/jez.2396. 32638552

[ref67] Telemeco RS , Gangloff EJ, Cordero GA, Mitchell TS, Bodensteiner BL, Holden KG, Mitchell SM, Polich RL, Janzen FJ (2016) Reptile embryos lack the opportunity to thermoregulate by moving within the egg. Am Nat 188: E13–E27. 10.1086/686628. 27322129

[ref68] Tollestrup K (1983) The social behavior of two species of closely related leopard lizards, *Gambelia silus* and *Gambelia wislizenii*. Z Tierpsychol 62: 307–320. 10.1111/j.1439-0310.1983.tb02159.x.

[ref69] US Drought Monitor (2021) California Percent Area in US Drought Monitor Categories. https://droughtmonitor.unl.edu/DmData/TimeSeries.aspx.

[ref70] Vicenzi N , Bacigalupe LD, Laspiur A, Ibargüengoytía N, Sassi PL (2021) Could plasticity mediate highlands lizards’ resilience to climate change? A case study of the leopard iguana (*Diplolaemus leopardinus*) in Central Andes of Argentina. J Exp Biol 224: jeb242647. 10.1242/jeb.242647. 34160050

[ref71] Warburg MR (1965) The influence of ambient temperature and humidity on the body temperature and water loss from two Australian lizards, *Tiliqua rugosa* (gray) (Scincidae) and *Amphibolurus barbatus cuvier* (Agamidae). Aust J Zool 13: 331–350. 10.1071/ZO9650331.

[ref72] Weaver SJ , Edwards H, McIntyre T, Temple SM, Alexander Q, Behrens MC, Biedebach RE, Budwal SS, Carlson JE, Castagnoli JO et al. (2022) Cutaneous evaporative water loss in lizards is variable across body regions and plastic in response to humidity. Herpetologica 78: 169–183. 10.1655/Herpetologica-D-21-00030.1.

[ref73] Weaver SJ , McIntyre T, van Rossum T, Telemeco RS, Taylor EN (2023) Hydration and evaporative water loss of lizards change in response to temperature and humidity acclimation. J Exp Biol 226: jeb246459. 10.1242/jeb.246459. 37767755

[ref74] Westphal MF , Stewart JAE, Tennant EN, Butterfield HS, Sinervo B (2016) Contemporary drought and future effects of climate change on the endangered blunt-nosed leopard lizard, *Gambelia sila*. PloS One 11: e0154838. 10.1371/journal.pone.0154838. 27136458 PMC4852947

[ref75] Wickham H (2022) tidyverse: Easily Install and Load the “Tidyverse.”

[ref76] Wickham H , Chang W, Henry L, Pedersen TL, Takahashi K, Wilke C, Woo K, Yutani H, Dunnington D (2022) ggplot2: create elegant data Visualisations using the grammar of graphics.

[ref77] Willmer P , Stone G, Johnston I (2005) Environmental Physiology of Animals, Ed2nd. Blackwell Publishing, Oxford, UK.

