# Supplementary material for: Hydric physiology and ecology of a federally endangered desert lizard
Source: Conserv Physiol. 2024 May 6;12(1):coae019. doi: 10.1093/conphys/coae019 (PMC11074591; doi:10.1093/conphys/coae019)
Supplement: Web_Material_coae019 [file web_material_coae019.pdf]

Appendix A: Hematocrit

Hematocrit is the percent red blood cells of blood; the remaining percent is mostly plasma, which is water based, so the relative quantity of hematocrit could indicate hydration state. We included hematocrit as a possible metric of hydric physiology because its values are easy to obtain, and it would be helpful to consider whether it is a unique, informative metric. When using microhematocrit capillary tubes to collect blood samples, it is simple and easy to record hematocrit after centrifuging and before separating out the plasma.

Plasma osmolality and hematocrit were closely related ($F_{1,114} = 15.1$, $p < 0.001$), suggesting that hematocrit is not a unique metric of hydric physiology when plasma osmolality is already being assessed. Like plasma osmolality, hematocrit had a negative relationship with CEWL ($F_{1,114} = 9.9$, $p = 0.002$). Hematocrit also had a positive relationship with body condition for female lizards, but no relationship for male lizards (direct effect: $F_{1,112} = 9.0$, $p = 0.003$; interaction effect: $F_{1,112} = 8.0$, $p = 0.005$), which could be due to the relationship between hematocrit and testosterone.

Appendix B: Water Supplementation

In an attempt to experimentally alter hydration via water supplementation and to investigate willingness of *G. sila* to drink water, we assigned radio-collared lizards to either water treatment (N=22, 11M:11F) or sham treatment (N=19, 10M:9F). Following measurements, lizards in the water treatment group were kept in plastic bins (42x27x16 cm) for ~1 hr with 2-3-inches of water in which they could easily stand and lean down to drink. Lizards in the sham treatment group were kept in the same bins, for the same amount of time, but with dry newspaper substrate to control for the effects of handling. For some water treatment lizards, we also tried dripping water into their mouths using a plastic dropper. The same handling was done for sham treatment lizards, but with no water. We recorded individual mass before and after water/sham treatment as a proxy for how much water individuals drank, if at all. To determine whether our supplemental hydration was successful, we ran linear regression and compared the model-predicted mean change in mass during the 1 hr hydration treatment for hydration versus sham lizards.

On average, lizards in the supplemental hydration group gained 0.37 g during treatment while lizards in the sham group lost 0.77 g ($t_{59} = -3.9$, $p = 0.0002$; $F_{1,59} = 15.5$, $p = 0.0002$), which amounted to average gains of 1% body mass for supplemental hydration lizards versus losses of 2% body mass during the treatment hour ($t_{59} = -3.7$, $p = 0.0005$; $F_{1,59} = 13.4$, $p = 0.0005$). However, we did not observe lizards drinking water in the treatment bins, and lizards refused to drink from the plastic dropper. There was no difference in the direction or amount of change in CEWL, plasma osmolality, or lizard mass throughout the study based on the hydration treatments given in April and May (all $|t| < 1$, $p > 0.4$). We also detected no differences in high

T_b, thermoregulatory accuracy, or microhabitat use based on supplemental hydration (all $|t| < 1.7$, $p > 0.1$). Thus, for our main text, the data for the different treatments were pooled.

These lizards should have been thirsty, due to it being a dry year, and based on their high plasma osmolality values. This is exactly why we attempted to give some lizards water, but *G. sila* seemed not to drink. Supplemental hydration treatment successfully led to a 1% increase in body mass, assumed to be from water intake, but it was apparently not enough to lead to measurable differences in hydric physiology or thermal ecology at later measurement periods. It is possible that our water supplementation treatments were too brief and stressful for lizards to relax and hydrate. However, we hypothesize that *G. sila* do not drink free-standing water at all in the wild, and that lapping dew droplets or eating water-rich arthropods would be more acceptable to these lizards and achieve a measurable difference in hydric physiology.
